# Cooperation and virulence in acute *Pseudomonas aeruginosa *infections

**DOI:** 10.1186/1741-7007-4-21

**Published:** 2006-07-07

**Authors:** Freya Harrison, Lucy E Browning, Michiel Vos, Angus Buckling

**Affiliations:** 1 Department of Zoology, University of Oxford, South Parks Road, Oxford OX1 3PS, UK; 2 Department of Zoology, University of Cambridge, Downing Street, Cambridge CB2 3EJ, UK; 3 Max Planck Institute for Developmental Biology, Spemannstrasse 35, 72076 Tuebingen, Germany

## Abstract

**Background:**

Efficient host exploitation by parasites is frequently likely to depend on cooperative behaviour. Under these conditions, mixed-strain infections are predicted to show lower virulence (host mortality) than are single-clone infections, due to competition favouring non-contributing social 'cheats' whose presence will reduce within-host growth. We tested this hypothesis using the cooperative production of iron-scavenging siderophores by the pathogenic bacterium *Pseudomonas aeruginosa *in an insect host.

**Results:**

We found that infection by siderophore-producing bacteria (cooperators) results in more rapid host death than does infection by non-producers (cheats), and that mixtures of both result in intermediate levels of virulence. Within-host bacterial growth rates exhibited the same pattern. Crucially, cheats were more successful in mixed infections compared with single-clone infections, while the opposite was true of cooperators.

**Conclusion:**

These data demonstrate that mixed clone infections can favour the evolution of social cheats, and thus decrease virulence when parasite growth is dependent on cooperative behaviours.

## Background

Numerous models of parasite evolution predict that mixed infections have higher virulence (host mortality rate) than do single-genotype infections. This is attributed to increased competition for host resources, favouring more rapid host exploitation [1-5]. There is, however, little empirical support for this hypothesis. A possible explanation for this lack of support is the assumption in many models that interactions between co-infecting strains are limited to simple resource competition, ignoring other inter-pathogen interactions. For example, competition can be mediated by the effects of the pathogens on the host immune system [6], by the production of anti-competitor toxins [7], and by one strain exploiting resources produced by the other [6,8-10]. Different predictions have been made regarding the impact of within-host parasite relatedness under these other forms of competition [8-11]. Of particular interest is the extent to which cooperative behaviour [8,9] will influence the virulence of mixed infections. Here, we experimentally address how parasite relatedness affects virulence of a bacterial pathogen when competition can result in one strain exploiting resource-scavenging molecules produced by another.

A major factor limiting the *in vivo *growth of parasitic bacteria is iron availability [12,13]. Under aerobic conditions, iron exists in the largely insoluble ferric form, and many host species actively withhold iron from infecting bacteria using high-affinity iron binding proteins [13]. In response, bacteria have evolved numerous mechanisms to scavenge iron from their hosts. One common mechanism is the production and uptake of siderophores, iron-scavenging agents released into the environment in response to iron deficiency. The relationship between siderophore production and bacterial growth rates has led to the belief that siderophore production enhances bacterial virulence; a view supported by the reduced virulence of mutants deficient in siderophore production [14-16].

A crucial feature of siderophore production is that it is a form of cooperation: siderophores potentially benefit all bacteria within the locality that are capable of taking up the siderophore, but are metabolically costly to the producer [8]. This makes siderophore production open to invasion by non-producing 'cheats', who pay none of the costs of siderophore production, but can still take up siderophores produced by nearby cells [8,9,17]. Kin selection theory predicts that bacteria are likely to produce siderophores (cooperate) when relatedness is high (i.e. when an infection is established by a single clone) [8,9,18-20]. This is because single-clone infections of cooperators will grow better and lead to more new infections than would single-clone infections of cheats; growth in the latter case is limited by poor iron uptake. By contrast, in a low-relatedness infection (when a single host is infected by multiple clones), cheats will be able to exploit co-infecting cooperator clones. This may afford a selective advantage to cheating. The reduction in total bacterial population growth rate resulting from the presence of cheats suggests that mixed-clone infections will be less virulent than single-clone infections [8,9].

We used the opportunistic pathogen *Pseudomonas aeruginosa *to test the hypothesis that siderophore cheats will have a selective advantage in mixed-clone compared with single-clone infections, and that their presence will reduce virulence. The primary siderophore of *P. aeruginosa *is the yellow-green pigment pyoverdine [13], allowing pyoverdine-negative 'cheat' colonies to be readily distinguished from wild-type 'cooperators' by their lack of yellow-green pigmentation [20,21].

## Results and discussion

**Figure 1:**
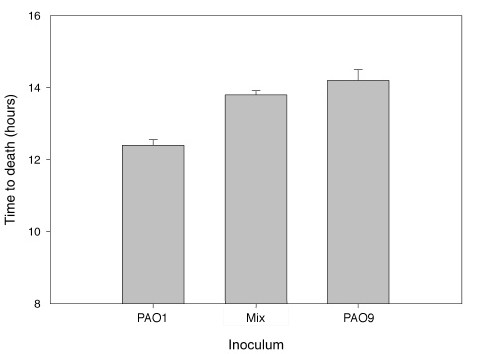
Time to death in hours of waxmoth larvae inoculated with pure cooperator, pure cheat or mixed clone infections of *P. aeruginosa*. Mixed inocula contained cheats and cooperators in a 1:1 ratio. Error bars show ± one standard error.

**Figure 2:**
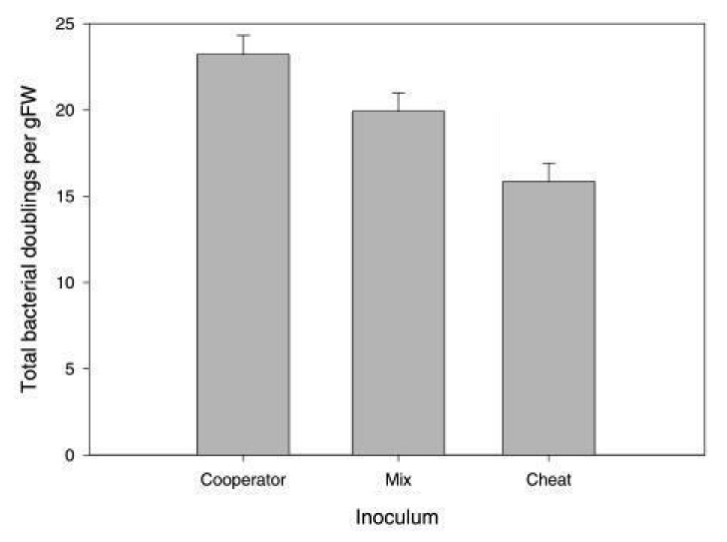
The effect of single versus mixed cooperator and cheat infections on the number of bacterial doublings (per gram fresh weight). Mixed inocula contained cheats and cooperators in a 1:1 ratio. Error bars show ± one standard error.

We inoculated waxmoth larvae (*Galleria mellonella*) [22] with single clones of a wild-type, pyoverdine-producing strain of *P. aeruginosa *(cooperator), an isogenic mutant of this strain that does not produce pyoverdine (cheat) [14], or both. We found that insects were on average killed by cooperator infections after 12 hours, 2 hours sooner than for cheat infections, and that mixed infections resulted in an intermediate time to death (Figure 1; Kruskal-Wallis test; *H *= 42.76, *P *< 0.0001; pair-wise comparisons (Mann-Whitney) showed significant differences between all groups: *P *< 0.01 in all cases). In separate experiments, we measured the growth rate of single- and mixed-clone infections over 8 hours, and this showed the same pattern as for virulence: cooperators grew faster than cheats, and mixed infections showed an intermediate growth rate (Figure 2, ANOVA: F_(2,55) _= 5.76, *P *= 0.005; pairwise comparisons: *P *^2 ^0.055). These data demonstrate that the presence of siderophore cheats can reduce the growth rate of a bacterial population, and hence reduce virulence.

**Figure 3:**
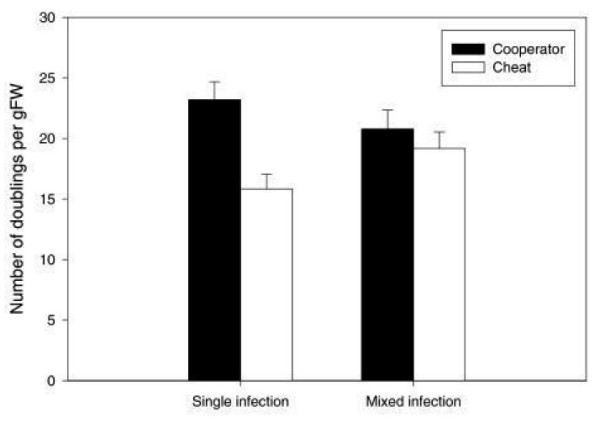
The number of doublings (per gram fresh weight) or cooperator (black) and cheat (white) clones in single and mixed infections. Mixed inocula contained cheats and cooperators in a 1:1 ratio. Error bars show ± one standard error.

We next addressed whether cheats were more likely to be favoured in mixed versus single-clone infections. As predicted, cheat populations grew more rapidly in mixed as opposed to single-clone infections, while the opposite pattern was observed for cooperators (Figure 3; ANOVA on log-transformed data shows a significant interaction between number of infecting clones and cheat/cooperator fitness; F_(1,75) _= 4.97, *P *< 0.03; main effects: cheat/cooperator fitness F_(1,75) _= 11.22, *P *< 0.002; number of clones F_(1,75) _= 0.26, *P *= 0.610). Mixed-clone infections are therefore more likely to favour the evolution of siderophore cheats than are single-clone infections, a result consistent with previous *in vitro *[20] and theoretical [8] work.

**Figure 4:**
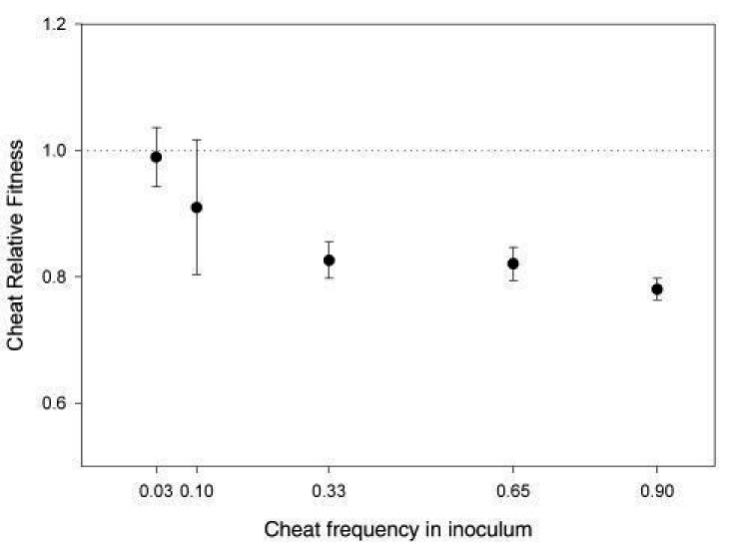
The relative fitness of the cheat in mixed infections decreases as its frequency in the inoculum increases. Error bars show ± one standard error.

We found that cheats had a slight selective disadvantage when in direct competition with cooperators at 1:1 ratios (Figure 3; relative fitness of cheats was less than 1: *t *= 2.30, *P *< 0.02). Both theory [19] and experiments on other microbial systems [23,24] suggest that the fitness of cheats should be a negative function of their frequency, as a rare cheat will have relatively more cooperators to exploit. To investigate frequency-dependent selection of siderophore cheats, we competed cooperators and cheats in caterpillars at a range of initial cheat frequencies (between 0.03 and 0.9). We found that the relative fitness of cheats increased as their initial frequency decreased (Figure 4; F_(1,33) _= 9.34, *P *< 0.005), such that the fitness of cheats and cooperators was not significantly different at cheat frequencies of 0.1 or lower (cheat relative fitness not significantly different from 1; *t *= 1.43, *P *= 0.19).

However, the relative fitness of cheats is noticeably lower in caterpillars than it is *in vitro *[20]; further *in vitro *assays carried out simultaneously with the *in vivo *work confirm this (data not shown). There are two likely explanations for this. The first is the greater spatial heterogeneity within a caterpillar compared with a media-filled tube. This is likely to increase the relatedness of immediate neighbours [19], reducing the chances for direct cooperator-cheat interactions and so bestowing a net benefit on cooperators. The second possible explanation is the longer periods of time bacteria spend at high densities in tubes compared with caterpillars; tube assays reached densities of approximately 10^8 ^cells/ml [20], while insect assays reached densities of approximately 10^7 ^cells/ml. The higher the population density of bacteria, the greater the likelihood that cheats will come into contact with cooperators [19,24]. Siderophore cheats have been observed at appreciable frequencies in chronic, clinical *P. aeruginosa *infections [17], and pyoverdine production has been known to decrease over the course of chronic infection [25]. These observations strongly suggest that cheats can enjoy a selective advantage in longer-term, high-density infections. The extent to which this apparent advantage is frequency dependent is not known. However, as most patients are initially colonised by a single, environmental clone [26-28], any cheats present will most likely have arisen within the patient and so must have invaded from an initially low frequency. We are currently carrying out studies investigating the *de novo* evolution of siderophore cheats over longer-term scales in this system to confirm this.

## Conclusion

Here, we have shown that mixed-clone infections of parasites can exhibit reduced virulence as a result of the breakdown of cooperative host exploitation. How competition between co-infecting parasites affects virulence in other circumstances will depend on the extent and modes of competition, and how parasites respond to this competition. However, cooperative interactions seem to be crucial to a range of bacterial traits associated with virulence, such as nutrient scavenging [14-16], the toxin-mediated breakdown of host tissues [29] and persistence (e.g. the formation of biofilms [30,31]). It is likely that mixed-clone infections will often impose selection for cheats and so show lowered virulence when virulence depends upon cooperation (see for example West and Buckling [8] and references therein). Furthermore, the introduction of 'cheating' genotypes to existing infections may provide a novel avenue to combat parasitic infections. While cheats remain lethal in our acute insect infection model, their ability to reduce virulence in this system suggests that they may have the potential to ameliorate the symptoms of localised, chronic bacterial colonisation in humans. It is clear that an expansion in our understanding of microbial community ecology may greatly improve existing models of virulence, and perhaps eventually suggest new, practical methods of treating microbial infections.

## Methods

### Bacterial strains and insect host

Strain PAO1 (ATCC 15692) was used as the wild-type pyoverdine producer. Our pyoverdine-negative strain was PAO9, derived from strain PAO6049 [32], a methionine auxotroph mutant of PAO1, using UV mutagenesis. Although PAO6049 requires exogenous methionine to grow, it has previously been shown [14] that when methionine is present, the virulence of PAO6049 does not differ significantly from that of PAO1. Thus, any observed differences in virulence between PAO1 and PAO9 may be attributed to pyoverdine production and not methionine auxotrophy. Strains were grown overnight on a rotary shaker at 37°C in 6 ml King's B medium to provide cultures for injection. Fifth instar waxmoth (*Galleria mellonella*) larvae (Livefood UK; ) were used as the insect host.

### *In vivo *virulence bioassay

Fresh overnight cultures of PAO1 and PAO9 were diluted in 0.8% NaCl solution. Larvae were randomly allocated to be inoculated with 10^2 ^colony-forming units (CFU) of PAO1, PAO9 or a 1:1 mixture of the two. Larvae were swabbed with 70% ethanol to prevent contamination of the injection site, and injected in the abdomen using a Hamilton syringe. The injection volume was 10 μl in all cases. Thirty larvae were assigned to each treatment. A further 30 larvae were injected with 10 μl of the NaCl solution as negative controls; their mortality rate was negligible. Larvae were incubated at 37°C and monitored for death at hourly intervals between 10 and 20 hours post-inoculation. Larvae were scored as dead if they failed to respond to mechanical stimulation of the head.

### Growth rate assays and competition experiments

Fresh overnight cultures of PAO1 and PAO9 were diluted in 0.8% NaCl solution. Two groups of 20 larvae each were swabbed with 70% ethanol and injected with 50 μl culture containing 0.5–1 × 10^4 ^CFU of either PAO1 or PAO9. A further group of 20 larvae was swabbed and injected with 0.5–1 × 10^4 ^CFU of PAO1 and PAO9 in a 1:1 mixture. Larvae were incubated at 37°C for 8 hours. Larvae were then weighed, dipped in 70% ethanol to kill surface contaminants, and homogenised in 500 μl M9 minimal medium using a plastic pestle. Homogenates were centrifuged at 3000 rpm for 3 minutes to pellet the solid material, and aliquots of diluted homogenate plated onto KB agar. Agar plates were supplemented with 15 μg/ml ampicillin to select against growth of native larval-gut bacteria (this concentration of ampicillin does not affect the growth of *P. aeruginosa*). Plates were incubated overnight at 37°C and numbers of PAO1 (green) and PAO9 (white) colonies scored. The relative fitness of PAO9 in the competition experiments was calculated from Malthusian growth parameters as described previously [33]. In mixed infections, the relative fitness was calculated by comparing the number of PAO9 doublings in each larva with the number of PAO1 doublings in the same larva. The fitness of PAO9 in single infections was calculated by comparing the number of PAO9 doublings in each larva with the mean number of single-infection PAO1 doublings. The fitness of PAO1 relative to PAO9 was calculated in the same manner. To investigate frequency-dependent fitness, *in vivo *competition experiments were carried out. The methodology for these experiments was the similar to that of the growth-rate assays, with the following modifications: groups of 10 larvae were injected with approximately 1–1.5 × 10^4 ^CFU of PAO1, PAO9, or mixtures containing 3, 10, 33, 65 or 90% PAO9, and incubated at 37°C for 18 hours.

## Authors' contributions

FH performed growth rate assays and competition experiments and drafted the manuscript; LEB carried out the in vivo virulence bioassay and contributed to the manuscript; MV and AB developed the use of waxmoth larvae as a model host for *P. aeruginosa*, optimised the assays used and advised during manuscript preparation. AB conceived of the study and helped to draft the manuscript. Statistical analyses were carried out by FH, LEB and AB.
